# Impact of COVID-19 Pandemic on the Diagnostic and Therapeutic Management of Endometrial Cancer: A Monocentric Retrospective Comparative Study

**DOI:** 10.3390/jcm12227016

**Published:** 2023-11-09

**Authors:** Francesco Plotti, Adele Silvagni, Roberto Montera, Carlo De Cicco Nardone, Daniela Luvero, Fernando Ficarola, Gianna Barbara Cundari, Francesco Branda, Roberto Angioli, Corrado Terranova

**Affiliations:** 1 Unit of Gynecology, Fondazione Policlinico Universitario Campus Bio-Medico, Via Alvaro del Portillo 200, 00128 Rome, Italy; 2 Unit of Medical Statistics and Molecular Epidemiology, Fondazione Policlinico Universitario Campus Bio-Medico, Via Alvaro del Portillo 200, 00128 Rome, Italy

**Keywords:** COVID19, pandemic, endometrial cancer, AUB (abnormal uterine bleeding), endometrioid EC, lymphadenectomy, sentinel lymph node

## Abstract

Endometrial cancer represents an ideal target to evaluate the impact of COVID-19 being the most frequent gynecological malignancy in Italy, generally detected at early stages and correlated with favorable oncological outcomes. The present comparative retrospective study carried out at Campus Bio-medico University Foundation in Rome aims to evaluate the impact of the COVID-19 pandemic on the presentation, diagnosis and treatment of EC. All women with a histological diagnosis of non-endometrioid and endometrioid endometrial cancer between 1 March 2018 and 31 October 2022 were included. The number of cases was higher in period 2 (95 vs. 64 cases). Time to diagnosis did not show statistically significant differences but in period 2, 92.06% of the diagnoses were made following abnormal uterine bleeding, while in period 1, only 67.02% were. The waiting time for the intervention was significantly shorter in period 2. Definitive histology, FIGO staging, surgical technique and adjuvant therapy did not show significant differences between the two periods. The study demonstrates that the impact of the COVID-19 pandemic did not have a direct effect on the diagnostic delay, tumor staging and type of therapy but rather on the presentation pattern of endometrial cancer.

## 1. Introduction

Endometrial cancer represents one of the most common gynecological neoplasms in industrialized countries; it is estimated that over 55,000 new cases are diagnosed each year in the United States [1,2,3]. One of the main indicators of survival and oncological outcomes is FIGO staging [4,5,6,7,8]. In fact, EC has a good prognosis at early stages, with a survival rate in Italy of 77% [9,10], thanks to its relatively early symptomatology: mainly abnormal vaginal bleeding (AUB). Transvaginal ultrasound examination allows the study of the endometrial rhyme, but the diagnostic test of choice is represented by hysteroscopy, which also allows for performing targeted biopsies for histological samplings [5,11]. The standard treatment for endometrial cancer is represented by simple hysterectomy with bilateral salpingo-oophorectomy and pelvic and para-aortic lymphadenectomy or evaluation of the sentinel lymph node which, in specialized centers, has replaced systematic lymphadenectomy [12,13,14,15]. The adjuvant treatment is then chosen on the basis of the tumor class of risk [16,17,18,19]. In the past two years, severe acute respiratory syndrome caused by Coronavirus 2 (SARS-CoV-2) has spread throughout the world. On 11 March 2020, WHO declared COVID-19 a pandemic [20]. The avoidance of needless therapies during the pandemic epidemic was advised by numerous guidelines in order to address the rising care needs of COVID-19 patients [21,22]. The WHO recommended strengthening health systems and reorganizing service delivery to respond to COVID-19, while maintaining basic essential services across the continuum of care, particularly in oncology [20]. To accommodate patients with COVID-19, hospitals in Italy have undergone reorganization, including setting up new spaces and critical care units [23]. Consequently, elective events have been rescheduled or canceled. This has partially translated into the risk of late diagnosis and treatment procrastination, with significant negative impacts on cancer patient outcomes [24]. The COVID-19 pandemic has actually hampered the timeliness of screening activities and routine checkups, principally delaying diagnostics and, as a result, the initiation of treatment [25,26,27,28,29]. The COVID-19 pandemic may be changing illness presentation patterns and threatening early access to care for individuals with malignancies, according to newly available data [30,31,32,33,34]. Endometrial cancer represents an ideal subject to assess the impact of COVID-19 for three main reasons: it is the most frequent gynecological neoplasm in Italy [9,10], it is generally detected at an early stage (72% of cases are diagnosed at FIGO stage I) [35], and when detected early, it typically correlates with positive oncological results [8,36,37,38]. 

The present study aims to evaluate the impact of the COVID-19 pandemic on the pattern of presentation, diagnosis and treatment of endometrial cancer to verify whether diagnostic and treatment strategies have changed across these two periods in an oncological center of reference at a territorial level. The primary endpoint aims to evaluate whether the diagnosis of endometrial cancer has been delayed due to the COVID-19 pandemic. The secondary endpoint aims to evaluate the variation of post-intervention therapeutic assistance in terms of time and type of therapy.

## 2. Materials and Methods

This is a single-center retrospective two-arm comparative study carried out at Campus Bio-Medico Foundation of Rome. All the patients diagnosed with endometrial cancer undergoing surgery between 1 March 2018 and 31 October 2022 were collected. Inclusion criteria: All women ≥18 years of age, diagnosed at primary or secondary discharge with malignant endometrial cancer, whose histologic diagnosis was endometrioid and non-endometrioid endometrial cancer (serous, mucinous, clear cell, or carcinosarcoma). All patients underwent the following preoperative work-up: clinical and gynecological examination, transvaginal ultrasound and magnetic resonance imaging (MRI) for preoperative assessment of myometrial and cervical stromal invasion depth. EC was diagnosed after histopathological examination of samples from operative hysteroscopy helpful for having a representative biopsy or for removal of the target lesion. An abdominal and thoracic computed tomography (CT) was considered for investigating the presence of extrapelvic disease in high-risk patients. All patients included have been collected into a database. On the basis of the date of surgery, the population was then split into two periods: period 1 includes patients undergoing surgery between 1 March 2018 and 29 February 2020, corresponding to the pre-pandemic era, while period 2 includes patients undergoing surgery between 1 March 2020 and 31 October 2022, corresponding to the pandemic era. The following data were collected into a database: medical history (BMI, age at the onset of menopause and at diagnosis), surgical history (date of hysteroscopy, date and type of surgery, type of lymphadenectomy if performed (sentinel vs. systematic), histological classification (definitive histological diagnosis and staging), clinical management (start date and type of adjuvant therapy if undertaken), time of diagnosis (days elapsed between the date of the first episode of abnormal vaginal bleeding (AUB) vs. date of suspect endometrial thickness at the ultrasound examination and the date of the histological diagnosis obtained with hysteroscopy), waiting time for surgery (days elapsed between the histological diagnosis and the date of the surgical intervention), initiation time of adjuvant therapy (days elapsed between the date of surgery and of the first day of any adjuvant therapy performed). The taxonomy proposed by WHO was used to classify the histological subtypes of endometrial cancer [36], while the FIGO criteria were used to determine the degree of differentiation and cytological atypia [8,37]. For surgical and adjuvant treatments, the indications dictated by the European guidelines ESMO/ESTRO/ESP were followed [38]. All patients underwent hysterectomy with bilateral salpingo-oophorectomy laparoscopically or laparotomically and did not have any macroscopic residual disease after surgery (RT = 0). Evaluation of lymph node status was systematically conducted in patients with non-endometrioid histology, FIGO IB or G3 disease. Lymph node evaluation was otherwise omitted in patients with endometrioid FIGO IA G1–G2 disease. Traditional clinic–pathological risk factors, especially age, histopathological type and grade, myometrial and LVSI invasion and molecular classification were adopted to assess the risk of recurrence and the indication for adjuvant therapy. A descriptive analysis was conducted of all the parameters listed above for the general population and separately for each of the two periods, specifically, in relation to the continuous variables, the mean (standard deviation) and the median (minimum-maximum) were calculated, in relation to the categories, the absolute and percentage frequencies were instead calculated. The distributive normality of the continuous variables was verified using the Shapiro–Wilk test. The differences between the 2 groups were investigated using the Mann–Whitney test (also known as the Mann–Whitney U test), since the continuous variables analyzed (e.g., BMI, age at diagnosis and age at onset of menopause) were not normally distributed, while the difference between the frequency distributions of categorical variables (e.g., nuclear grade, FIGO classification, etc.), by chi-square/Fisher test. Differences between groups were investigated using the Mann–Whitney test (also known as the Mann–Whitney U test). Where appropriate, the ratio between the risks of the two groups of patients (OR) was calculated. All tests were scored with α = 0.05. The analyses were performed using Excel version 16.49 and R version 4.0.2. The study was conducted in accordance with the Declaration of Helsinki and approved by the Institutional Review Board of Foundation University Campus Bio-Medico (protocol code PAR 62.22, on Monday 31 October 2022).

## 3. Results

The general population is composed of 159 women aged between 33 and 87 years who underwent surgery for endometrial cancer at Campus Bio-medico Foundation between 1 March 2018 and 31 October 2022. In period 1, pre-pandemic, a total of (64) cases were subjected to surgery, while in period 2 (95). 

The demographic characteristics of the population sample under analysis are reported in Table 1.

The average time to diagnosis is 139 days. In period 1, the mean time to diagnosis was 131 days, while in period 2 it was 142 (*p*-value = 0.62414). However, these data are attributable only to those cases who underwent hysteroscopy following abnormal vaginal bleeding, 43 cases (67.2%) in period 1 and 88 cases (92.6%) in period 2, for a total of 131 cases (82.4%) in the general population. In parallel, 28 patients (17.6%) in the general population, distributed in 21 patients (13.2%) in period 1 and 7 patients (4.4%) in period 2, achieved histological diagnosis of endometrial cancer despite not having symptoms but only due to periodic specialist visits. The mean waiting time for the intervention from the general population was 62 (59) days. In period 1 it was 79 (83) days, while in period 2, it was 49 (31) days (*p*-value = 0.01314). The waiting time for the intervention was therefore significantly different in period 2 compared to period 1.

The time to adjuvant therapy initiation of the general population averaged 89 (59) days, specifically in period 1 it was 79 (48) days, and in period 2 it was 96 days (66) (*p*-value = 0.08186). The mean time to adjuvant therapy initiation was therefore significantly different in period 2 compared to period 1. All the data reported are summarized in Table 2.

As reported in Table 3, the histological data show a prevalence of endometrioid histo-type endometrial cancer in the general population and in each single period with 135 cases (84.91%) in the general population, 54 cases (84.37%) in period 1 and 81 cases (85.26%) in period 2 (*p*-value = 1). 

Data regarding the FIGO classification show a clear prevalence of endometrial cancer in FIGO stage I: 124 cases (77.99%) among the general population, divided into 49 cases (76.57%) in period 1 and 75 cases (78.95%) in period 2, as reported in Table 4.

The surgical technique most commonly used in the general population was the laparotomy (LPTM) with 101 (63.52%) total laparotomic interventions, while the laparoscopic technique (LPS) was preferred in 36.48% (58 cases) of interventions. In period 1, the total laparoscopic operations were 19 (29.68%) while the laparotomic ones were 45 (70.32%). In period 2, laparoscopic operations were 39 (41.05%) while laparotomic operations were 56 (58.95%). During surgery, the sentinel lymph node technique was used in 23.90% (38 cases) of cases of the general population, especially in 21.87% (14 cases) of the surgical interventions of period 1 and in 25.26% (24 cases) of period 2 operations. Systemic lymphadenectomy was preferred in 82 (51.57%) operations of the general population, divided into 29 (45.31%) cases in period 1 and 53 (55.79%) cases in period 2. As reported in Table 1, no statistically significant differences were found regarding three surgical techniques (sentinel lymph node vs. systemic lymphadenectomy vs. no lymphadenectomy) adopted in the two periods analyzed. All the data reported are summarized in Table 5.

As reported in Table 6, out of the whole population, 77 patients (48.43%) underwent adjuvant therapy; among these, 19 (11.95%) underwent exclusive chemotherapy, 23 (14.46%) exclusive radiotherapy and 35 (22.01%) radio-chemotherapy. As reported in Table 1, no statistically significant differences were found regarding the adjuvant therapies adopted in the two periods.

**Figure 1:**
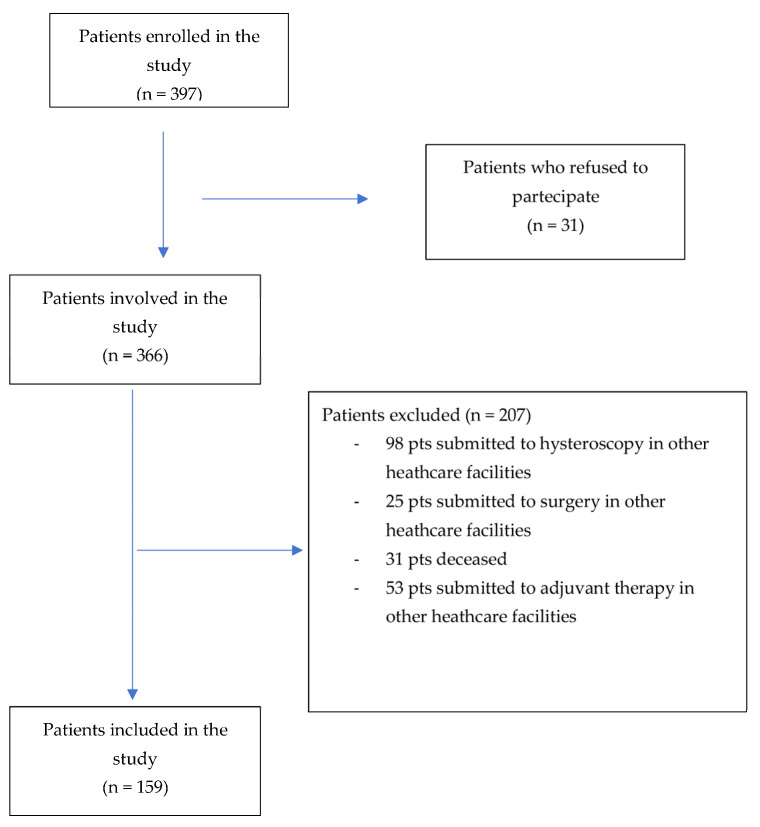
Study flow chart.

Flow chart of the study is shown in Figure 1.

## 4. Discussion

The present study aimed to investigate the management of patients with endometrial cancer before and during the COVID-19 pandemic. Compared to other studies in the literature [39,40,41,42], which investigated the impact of the pandemic on the diagnosis and therapy of endometrial cancer in the first months of the pandemic itself, the present study compares the two years prior to the pandemic (period 1) with the two years during the pandemic (period 2) to assess the impacts over a longer time span. By attempting to account for the associated long-term impacts, our retrospective two-arm comparative study enables a more in-depth investigation of the pandemic impact on endometrial cancer patterns of care.

Considering the BMI and age at diagnosis, no statistically significant differences were found between the two periods, i.e., period 1 (pre-pandemic), and period 2 (during the pandemic), while there is sufficient statistical evidence to state that there is a real difference in the ages of menopause onset among the two groups. This may be attributable to the physiological variation of EC incidence over time.

Beginning with the total number of patients gathered at the Campus Bio-Medico Foundation of Rome, we discovered that period 2 had more patients than period 1 (95 vs. 64 cases). These data are justifiable by the fact that our foundation became a point of reference during the pandemic for all patients who were not able to be treated in other facilities reorganized in favor of COVID-19 management, with limited admissions and assistance in other departments. These data are in contrast with what has been reported by Bogani et al. [40], where it is shown that fewer EC overall cases received medical and/or surgical care during the pandemic. In contrast with the data reported in the literature, not only did we not record a decrease, but rather we pointed out a statistically significant increase in the total cases of EC accessed to our facility.

All three intervals evaluated (diagnosis time, waiting time for surgery and time to start adjuvant therapy) show positive asymmetry in distribution, moreover, the mean values (more susceptible to extreme values) are greater than the median values; this suggests that in the general population, as well as within the two groups, long waiting periods are less frequent than short waiting periods.

As the principal theme of evaluation, being the main endpoint of the study, time to diagnosis did not show statistically significant differences between the two intervals. This is in contrast to what we would have expected given the reduction in hospital and routine outpatient visits, as reported in the literature, in order to avoid crowding and therefore the spread of the infection [31,43,44,45,46,47]. However, the present study highlighted significant differences regarding the diagnostic method. In fact, in period 2 about 92% of the diagnoses were made following AUB, while in period 1 only 67% were. From these data, it can be deduced that access to routine specialist visits during period 2 was reduced and limited to those cases in which the patient experienced an “alarm symptom” to require a diagnostic investigation. Therefore, even if no significant diagnostic delay was evidenced, defined as days elapsed between the first bleeding episode/first ultrasound endometrial thickness evidence and the diagnosis, the greater frequency of diagnoses obtained after the symptom is an indirect index of a longer wait to perform check-ups and hence a delayed diagnosis. Although the presence of symptoms does not correlate with the severity of the pathology [36,48,49,50], these data underlines how important check-ups and prevention are in order to obtain an early diagnosis for those tumors, such as endometrial carcinoma, for which there is no existing screening method.

Compared to the multicenter study conducted by Bogani et al. [40], and in contrast with the literature, which did not demonstrate any discernible variations in the period between diagnosis and surgical therapy, in our study the waiting time for surgery was significantly different in period 2 compared to period 1, with an average waiting period, respectively, of 49 and 79 days. In fact, during the pandemic, health institutions asked healthcare facilities to postpone elective and deferrable surgeries in favor of urgent and oncological procedures. As a result, in the present study, it was determined that the pandemic period’s waiting time was shorter than the pre-pandemic one with statistical significance of the reported value. These data are probably related to the absence of the entire elective, and therefore deferrable, component of surgery, making it possible to streamline the operating lists for oncological surgery. The net result is a reduction in surgical waiting times for malignant pathologies.

The timing of initiation of adjuvant therapy does not statistically differ between the two groups, for this reason, there was no evidence of longer waiting times to access adjuvant therapies during the pandemic compared to the previous period. All this is in agreement with what was reported by Bogani et al. [40] and is partly justifiable by the effective reorganization of services aimed at managing a greater number of patients due to the increase in the oncological care burden of our facility, which became a territorial reference point.

As reported in the guidelines [36,49,51,52,53], the most widely used surgical technique was laparotomy with no significant differences in the surgical technique adoption (laparoscopy vs. laparotomy) evidenced between the two periods under analysis, in agreement with what is reported in the literature [42,54,55,56].

In parallel, in the present study, the type of lymphadenectomy (systematic vs. sentinel) did not show significant differences between the two groups. These data are in contrast to what was reported by Bogani et al. [40], who pointed out an increase in cases in which the sentinel node technique and backup lymphadenectomy were applied. The above may be justified by the fact that in the present study, the definitive histology, nuclear grading and the FIGO staging of the tumor did not show significant differences between the pandemic and pre-pandemic periods. These data, in fact, differ from those present in the literature, which discloses a higher incidence of non-endometrioid type and major stage tumors during the pandemic [40,50,57,58] and may be partially explained by the lack of diagnostic delay and rapidity of the therapeutic proposal of our center.

Finally, in relation to our secondary endpoint, focused on the investigation of the modifications in terms of post-surgical treatments, there are no statistically significant differences between period 1 and period 2 in terms of types of adjuvant therapy undertaken. In contrast to the evidence of the study by Bogani et al. [40], there was no evidence of an increase in cases requiring adjuvant therapy in the pandemic period compared to the pre-pandemic. These data are in line with the results previously discussed, since the choice of the type of adjuvant therapy is based on the risk class [36,59,60] and since there were no variations in terms of definitive histology and FIGO staging (main variables for determining the risk class). 

A 2018 Israeli Gynecologic Oncology Group retrospective study evaluated the role of EC diagnosis in asymptomatic patients [48]. The authors compared data from 1374 patients presenting with postmenopausal bleeding with 233 asymptomatic patients. Although EC identified in asymptomatic postmenopausal females is not related to a survival advantage, it has been found that asymptomatic patients had a lower proportion of cases with more advanced disease stages and a lower rate of adjuvant medication administration. Based on the findings of this investigation, it would be interesting to find out how the various endometrial cancer presenting patterns discovered during the pandemic may affect patients’ survival and oncological outcomes [61]. Our data, which showed no impact of these data on FIGO staging and grading of tumors, should be applied to a larger sample of the population to corroborate or disprove the results.

The study demonstrates that the impact of the COVID-19 pandemic did not have a direct effect on the delay in diagnosis but rather on the pattern of presentation of endometrial cancer. Therefore, in pursuing our primary endpoint, which consisted of the evaluation of whether a diagnostic delay had occurred in the pandemic phase compared to the pre-pandemic one, we verified that no statistically significant delay was recorded. However, a change in the presentation pattern of the disease and in the investigation method through which the initial clinical suspicion was raised were highlighted. In fact, patients came to our attention more frequently with endometrial cancer symptoms, primarily abnormal vaginal bleeding, as a result of the decreased access to regular gynecological checkups, rather than after an ultrasound examination evidencing an increased endometrial thickness.

The main limitations of the present study, which could be otherwise interesting starting points for future investigations are the following: the limited number of samples in the exam and, due to the absence of long-term follow-up, the lack of evaluation of the impact of the COVID-19 pandemic on the oncologic outcomes of EC patients involved. However, the aim of this research was to assess how the pandemic impacted patients’ access to care and endometrial cancer pattern of presentation rather than to determine the impact the pandemic produced on survival and oncological outcomes. Further studies may have to assess how waiting time for diagnosis and treatment and different patterns of presentation may have impacted survival outcomes, but a more prolonged follow-up is certainly needed to determine aspects of this relevance.

The main strengths of this paper consist of a data analysis that includes a very long time span (4 years) for the first time in the literature on this topic and carrying out the study in one of the few centers in Italy that has not undergone substantial reconditioning for the management of the pandemic crisis, becoming a first-level oncological center of reference at a territorial level. A further point of strength is related to the large number of patients for whom it was possible to reconstruct the entire process (from diagnosis to adjuvant treatment completed in our facility) and therefore precisely calculate the various timescales in the exam.

## 5. Conclusions

In conclusion, the present study shows that the characteristics and patterns of care are changing during the COVID-19 pandemic, highlighting that the diagnosis was made mainly as a result of alarm symptoms, rather than for evidence during routine gynecological visits, and despite this, no diagnostic delay was detected. Conversely, waiting times for surgery were reduced and no impacts on FIGO staging, tumor grading, need for and type of adjuvant therapy were found compared to the pre-pandemic era.

The Campus Bio-Medico Foundation Hospital has therefore been able to maintain excellent therapeutic assistance for patients with endometrial cancer during the COVID-19 pandemic, reducing waiting times for diagnosis and surgery and guaranteeing the start of adjuvant therapy comparable to previous years. These aspects are related to the fact that the center is a first-level oncological center of reference at a territorial level.

Further studies, on a larger sample, will be needed to translate what has been highlighted in terms of survival and oncological outcomes in order to demonstrate if and how the pandemic has changed long-term outcomes.

## Figures and Tables

**Table 1:** Population demographic characteristics.

	Total	Interval 1	Interval 2	*p*-Value
n. patients	159	64	95	0.9822
Age (mean)	64.89 (10.28)	64.36 (11.05)	65.24 (9.77)	0.9442
BMI (mean)	29.94 (11.78)	28.85 (6.23)	30.68 (14.35)	0.5892
Menopause onset (mean age)	50.81 (±3.61)	50.07 (3.94)	51.27 (3.34)	0.03752

**Table 2:** Histologic characteristics.

Histology	Total	Interval 1	Interval 2	*p*-Value
ENDOMETRIOID G1	159	64	95	0.9822
ENDOMETRIOID G2	64.89 (10.28)	64,36 (11.05)	65.24 (9.77)	0.9442
ENDOMETRIOID G3	29.94 (11.78)	28.85 (6.23)	30.68 (14.35)	0.5892
NON ENDOMETRIOID	50.81 (±3.61)	50.07 (3.94)	51.27 (3.34)	0.03752
HYSTOLOGY				

**Table 3:** Mean waiting time.

	Total	Interval 1	Interval 2	*p*-Value
Time to diagnosis (mean days)	139	131	142	0.62414
Time to surgery (mean days)	62	79	49	0.01314
Time to adjuvant therapy (mean days)	89	79	96	0.08186

**Table 4:** Surgical technique.

Surgical Technique	Total	Interval 1	Interval 2	*p*-Value
N. Laparotomies	101 (63.52%)	45 (70.32%)	56 (58.95%)	0.1963
N. Laparoscopies	58 (36.48%)	19 (29.68%)	39 (41.05%)	0.1963
N. SENTYNEL LYMPHNODE	38 (23.90%)	14 (21.87%)	24 (25.26%)	0.7628
N. SYSTEMIC LYMPHADENECTOMY	82 (51.57%)	29 (45.31%)	53 (55.79%)	0.2565
N. NO LYMPHADENECTOMY	39 (24.53%)	21 (32.82%)	18 (18.95%)	0.3442

**Table 5:** FIGO staging.

Figo Staging	Total	Interval 1	Interval 2	*p*-Value
STAGE I	124 (77.99%)	49 (76.57%)	75 (78.95%)	0.8722
STAGE II	11 (6.92%)	5 (7.81%)	6 (6.32%)	0.7568
STAGE III	20 (12.58%)	10 (15.62%)	10 (10.53%)	0.3434
STAGE IV	4 (2.52%)	0	4 (4.21%)	0.1489

**Table 6:** Adjuvant therapy.

Adjuvant Therapy	Total	Interval 1	Interval 2	*p*-Value
CMT	19 (11.95%)	11 (17.19%)	8 (8.42%)	0.1336
RT	23 (14.46%)	7 (10.94%)	16 (16.84%)	0.3623
CMT + RT	35 (22.01%)	13 (20.31%)	22 (23.16%)	0.8184

## Data Availability

Data are unavailable due to privacy.
